# Algae-fungi symbioses and bacteria-fungi co-exclusion drive tree species-specific differences in canopy bark microbiomes

**DOI:** 10.1093/ismejo/wrae206

**Published:** 2024-10-17

**Authors:** Jule Freudenthal, Kenneth Dumack, Stefan Schaffer, Martin Schlegel, Michael Bonkowski

**Affiliations:** Terrestrial Ecology, Institute of Zoology, Cluster of Excellence on Plant Sciences (CEPLAS), University of Cologne, Zülpicher Str. 47b, Köln 50674, Germany; Terrestrial Ecology, Institute of Zoology, Cluster of Excellence on Plant Sciences (CEPLAS), University of Cologne, Zülpicher Str. 47b, Köln 50674, Germany; Molecular Evolution and Systematics of Animals, Institute of Biology, University of Leipzig, Talstraße 33, Leipzig 04103, Germany; Biodiversity and Evolution, Institute of Biology, University of Leipzig, Talstraße 33, Leipzig 04103, Germany; German Centre for Integrative Biodiversity Research (iDiv) Halle Jena Leipzig, Puschstraße 4, Leipzig 04103, Germany; Terrestrial Ecology, Institute of Zoology, Cluster of Excellence on Plant Sciences (CEPLAS), University of Cologne, Zülpicher Str. 47b, Köln 50674, Germany

**Keywords:** forest ecosystems, microbial diversity, host specificity, eukaryotes, prokaryotes, lichen, plant holobiont

## Abstract

With over 3 trillion trees, forest ecosystems comprise nearly one-third of the terrestrial surface of the Earth. Very little attention has been given to the exploration of the above-ground plant microbiome of trees, its complex trophic interactions, and variations among tree species. To address this knowledge gap, we applied a primer-independent shotgun metatranscriptomic approach to assess the entire living canopy bark microbiome comprising prokaryotic and eukaryotic primary producers, decomposers, and various groups of consumers. With almost 1500 genera, we found a high microbial diversity on three tree species with distinct bark textures: oak (*Quercus robur*), linden (*Tilia cordata*), both with rough bark, and maple (*Acer pseudoplatanus*) with smooth bark. Core co-occurrence network analysis revealed a rich food web dominated by algal primary producers, and bacterial and fungal decomposers, sustaining a diverse community of consumers, including protists, microscopic metazoans, and predatory bacteria. Whereas maple accommodated a depauperate microbiome, oak and linden accommodated a richer microbiome mainly differing in their relative community composition: Bacteria exhibited an increased dominance on linden, whereas co-occurring algae and fungi dominated on oak, highlighting the importance of algal-fungal lichen symbioses even at the microscopic scale. Further, due to bacteria-fungi co-exclusion, bacteria on bark are not the main beneficiaries of algae-derived carbon compounds as it is known from aquatic systems.

## Introduction

Forests cover almost one-third of the terrestrial surface of the Earth, with more than 3 trillion trees worldwide [1, 2]. They form the primary interface between terrestrial biomes and the atmosphere, of which bark surfaces, the rhytidome, constitute a huge and important part [3, 4]. However, microbial pathogens, such as *Oomycota* (e.g. Sudden Oak Death, Lime Disease) or fungi (e.g. Sooty Bark Disease of maple), are increasingly threatening forest ecosystems [5, 6]. Thus, a deeper knowledge of the diversity and composition of what might be considered a “healthy microbiome” of forest trees is urgently needed.

Tree bark surfaces provide long-lasting habitats colonized by algae and cyanobacteria as primary producers, prokaryotes and fungi as decomposers, and predatory bacteria, heterotrophic protists, and microscopic metazoans as consumers. They collectively form a complex microbial food web—the bark microbiome. Bark microbiomes may differ between tree species, due to substantial differences in the physical surface structures and chemical characteristics of tree barks [7, 8]. For example, rough bark may retain humidity and nutrients better than smooth bark and may provide increased protection against environmental stressors such as UV radiation and wash-off by rainfall [9, 10]. Primer-based DNA 16S, ITS, and 18S rRNA gene amplicon sequencing studies of selected microbial taxa indicated tree species-specific differences for bacteria and fungi on bark [9, 11, 12], but not for protists [13]. There are also indications that the bark structure influences the composition of algae and microscopic metazoan communities [14, 15]. However, no study has yet comprehensively analyzed the entire canopy bark microbiome and its potential trophic interactions [7].

So how diverse are the entire bark microbiomes among tree species and are they shaped by differences in bark surface texture? Using a primer-independent shotgun metatranscriptomic approach, the present study analyzes the diversity and composition of the bark microbiomes of three tree species in a floodplain forest. The strength of such an approach lies in the simultaneous assessment of the entire diversity of prokaryotes and eukaryotes [16] and avoiding the selectivity and biases inherent to primer-based methods, in particular the strong biases associated to universal eukaryotic primers [17, 18]. Additionally, metatranscriptomics have shown to be more accurate than metagenomics for the taxonomic identification of microbial communities at equal sequencing depths [19]. Further, this RNA-based approach mainly targets living microorganisms and is therefore rather insensitive to dead microorganisms and environmental DNA [20, 21] which can be highly enriched on tree bark [22]. This approach allowed, to (i) comprehensively investigate the entire bark microbial and microfauna food web of canopies using one single methodology and dataset, (ii) explore potential relationships between primary producers, decomposers and consumers in these food webs, and (iii) analyze differences between microbial communities of different tree species. We hypothesized that (i) bark microbiomes of different tree species would differ and that (ii) microbial communities on the two tree species with rough bark surfaces pedunculate oak (*Quercus robur*) and small-leaved linden (*Tilia cordata*) would be more similar than those on sycamore maple (*Acer pseudoplatanus*) with a distinctly smoother bark texture.

## Materials and methods

### Sampling, RNA extraction and sequencing

Bark samples in tree canopies were collected on May 23, 2022, in the Leipzig floodplain forest (51.3657 N, 12.3094 E) in Germany in cooperation with the Leipzig Canopy Crane Facility. Five different tree canopies were sampled, for each of three tree species respectively: *Quercus robur*, *Tilia cordata*, and *Acer pseudoplatanus*. Samples were taken at the average mid-canopy height of 23 ± 3.5 m. To further reduce the influence of spatial variation in canopies, accessible branches at all four celestial directions were sampled by scraping off the biocrust on the bark using a sterile scalpel blade. Special care was taken to avoid any macroscopic organisms, including any visible lichen or mosses. The material was collected in sterile tubes (SARSTEDT AG & Co. KG, Nümbrecht, Germany), immediately placed on dry ice, and subsequently stored at −80°C until RNA extraction.

RNA was extracted from 0.2 g of the collected material with the RNeasy PowerSoil Total RNA Kit (Qiagen GmbH, Hilden, Germany) according to the instructions of the manufacturer, except for the disruption step, which was carried out on a FastPrep-24 homogenizer (MP Biomedicals, Eschwege, Germany) for 30 s at 5.5 m s^−1^. Subsequently, the RNA was eluted in 90 μl buffer SR7, directly followed by DNA digestion using Ambion Dnase I (Thermo Fisher Scientific Inc, Darmstadt, Germany) and RNA purification using the MEGAclear kit (Thermo Fisher Scientific Inc). RNA concentrations were quantified by Qubit RNA High sensitivity Assay-Kit (Thermo Fisher Scientific Inc) and Qubit 4 Fluorometer (Thermo Fisher Scientific Inc).

For library preparation, the NEBNext Ultra II Directional RNA Library Prep Kit for Illumina (New England Biolabs, Ipswich, MS, USA) was used with 100 ng total RNA and without the depletion of ribosomal RNAs or selection of mRNAs. Sequencing was performed on a NovaSeq Sequencing System (Illumina Inc., San Diego, CA, USA) with a paired-end sequence length of 150 bp at the Cologne Center for Genomics (Köln, Germany).

### Sequence processing

After quality assessment of the raw data using FastQC v. 0.11.9 [23], quality filtering was performed using FastP v 0.23.2 [24]. All read pairs that contained any low-quality base <10% or had more than 10% bases with a quality score of <25 were excluded. Furthermore, adapter sequences and read pairs with low complexity or ambiguous bases were removed (Supplementary Table 1). Paired-end reads were assembled into contigs using Mothur v. 1.48.0 [25]. Subsequently, all contigs were filtered for a minimum contig length of 100 bp without ambiguities or mismatches and a minimum overlap of 10 bases (Supplementary Table 1). Additionally, the proportion of messenger RNA (mRNA) in the samples was estimated using SortMeRNA v. 4.3.4 [26], which, on average, accounted for only 4.2% of the total RNA.

For the taxonomic assignment BLASTN v. 2.10.0 [27] was employed to compare the sequences against the SILVA 138 SSU Ref Nr. 99 database for the prokaryotes and the PR^2^ database v. 4.14.0 [28] for the eukaryotes. The sequences were filtered by an *E* value threshold of 1e^−50^ keeping only the best match (Supplementary Table 1). However, considering the limitations of the sequencing method, i.e. sequencing of random fragments with a length of 150 bp, a very conservative taxonomic assignment at genus level was performed (similarity threshold of ≥85%) to avoid an overestimation of the microbial diversity [29, 30]. Further, genera with proportional reads below 0.001% across all samples as well as putative contaminants were excluded, such as macroscopic animals, land plants (*Embryophyceae*), and chloroplasts. Fungi were classified based on the taxonomic assignment into functional groups as lichen-forming fungi, (facultative) yeasts, and plant parasites [31], and lichen-forming algae were identified according to a previously published database [32]. Because the vast majority of organisms were microbial, we will refer to the assessed communities as “microbial communities”, although they also included microscopic metazoans, i.e. rotifers, tardigrades, and nematodes. Further, we will refer to the prokaryotic community as “bacterial community”, as *Archaea* represented on average only 0.002 ± 0.004% of the prokaryotic community.

### Statistical analyses

All statistical analyses and data visualizations were carried out in R v. 4.3.1 [33] with the packages ggalt v. 0.4.0, ggplot2 v. 3.4.2, ggpubr v. 0.6.0, ggrepel v. 0.9.3, ggsignif v. 0.6.4, and ggthemes v. 4.2.4 [34–39]. Rarefaction curves were calculated from count data (vegan v. 2.6–4::rarecurve [40]) to check whether the sampling effort covered the taxonomic diversity (Supplementary Fig. 1). As all rarefaction curves reached complete saturation, data analysis was proceeded without normalizing for sequencing depth (Supplementary Fig. 1).

The relative proportion of the 10 most abundant classes of bacteria, algae, fungi, heterotrophic protists, and microscopic metazoans that constituted more than 1% of the respective community as well as the 10 most abundant genera classified to genus level was visualized by Sankey diagrams (riverplot v. 0.10::makeRiver [41]). Core co-occurrence network analyses were conducted using Sparse and Compositionally Robust Inference of Microbial Ecological Networks [42]. This method considers the compositionality of the data and reduces indirect edges by using sparse neighborhood or inverse covariance selection. To improve the robustness of the analysis, only genera accounting for more than 0.01% across all samples and present in all samples were included, as co-absence may yield spurious edges [43]. The remaining genera were combined into a pseudo-taxon. The sparse Meinshausen-Buhlmann’s neighborhood selection method, with a lambda.min.ratio = 0.001 and nlambda = 100 (package SpiecEasi v. 1.1.2) was used for network inference. The number of associations between the genera was summarized for functional trophic and taxonomic groups, i.e. algae and cyanobacteria (primary producers), fungi and other bacteria (decomposers), and predatory bacteria, protists and microscopic metazoans (consumers), showing the ratio of positive to negative associations. The core co-occurrence network was visualized using Cytoscape v. 3.9.0 [44].

To compare alpha and beta diversities of microbiomes between tree species, we considered the whole community as well as the 10 most abundant classes (fungi and microscopic metazoans) or domains (protists and algae) of the eukaryotes and the 10 most abundant phyla of the bacteria represented by at least 10 genera (referred to as microbial groups). Alpha diversity was assessed based on genus richness and Pielou’s Evenness index using the functions vegan v. 2.6–4::diversity and vegan v. 2.6–4::specnumber [40]. Alpha diversity indices among tree species were compared by using a Kruskal–Wallis test and Wilcoxon post-hoc test (rstatix v. 0.7.2::kruskal_test and rstatix v. 0.7.2::wilcox_test [45]), corrected for multiple comparisons [46]. To visualize shifts in microbial community compositions in relation to tree species, non-metric multidimensional scaling (NMDS) based on Bray–Curtis dissimilarity of relative abundances was conducted (vegan v. 2.6–4::metaMDS [40]). Differences in community composition between tree species were tested by a permutational multivariate analysis of variance (perMANOVA, vegan v. 2.6–4::adonis2 [40]) and the function pairwise.adonis2 for pairwise comparisons (pairwiseAdonis v. 0.4.1 [47]). In addition, we performed NMDS for algae, bacteria, fungi, and protists, respectively, and correlated the relative abundance of the microbial groups as well as of the respective 10 most abundant genera that could be classified to the genus level onto the ordination (vegan v. 2.6–4::envfit [40]), only significant (*P* value <0.05) correlations are shown.

## Results

### Overall microbial diversity of bark surfaces

**Figure 1:**
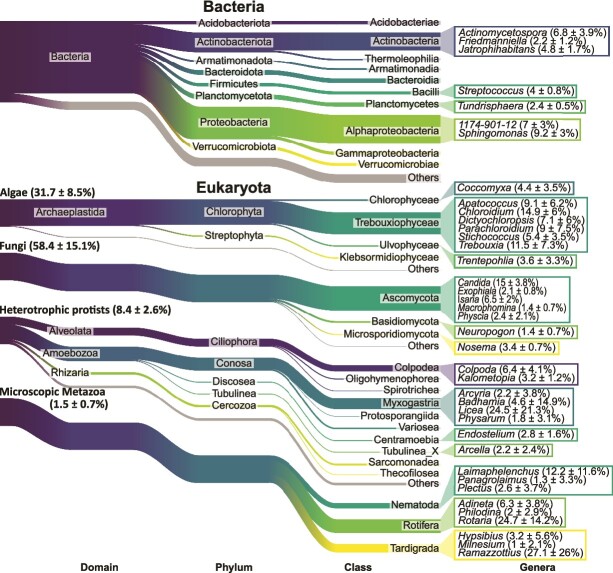
**Microbial community composition in tree canopies.** Sankey diagrams with the mean (± 1SD) relative abundance (%) across all tree species (N = 15) showing the 10 most abundant classes of bacteria, algae, fungi, heterotrophic protists, and microscopic metazoa that accounted for more than 1% of the respective community. Additionally, the 10 most abundant genera that were classified to genus level are shown for each community, respectively.

We obtained 16 ± 1.5 million ribosomal RNA sequences per sample, revealing altogether a microbiome diversity of almost 1500 genera from 28 algal, 53 bacterial, nine fungal, 33 protistan, and three microscopic metazoan classes on the tree bark surfaces (Fig. 1, Supplementary Fig. 2). On average, 60.3 ± 9.4% of these sequences could be assigned to in total 645 bacterial genera and 39.7 ± 9.4% to 113 algal, 558 fungal, 155 protistan, and 16 microscopic metazoan genera. Algae as primary producers accounted for 31.7 ± 8.5% of the eukaryotic reads, whereas cyanobacteria represented only a minor fraction (0.2 ± 0.1% of bacterial reads). Accordingly, primary producers were dominated by green algae in *Trebouxiophyceae* (83.2 ± 30.8% of algal reads), with dominant genera such as *Chloroidium*, *Trebouxia*, and *Apatococcus* (14.9 ± 6, 11.5 ± 7.3, 9.1 ± 6.2% of algal reads, respectively) as well as *Coccomyxa* (*Chlorophyceae*, 4.4 ± 3.5% of algal reads) and *Trentepohlia* (Ulvophyceae, 3.6 ± 3.3% of algal reads). Among decomposers, by far the most abundant bacteria were *Alphaproteobacteria* (34.9 ± 5.2% of bacterial reads), dominated by *Sphingomonas* accounting for nearly 10% of the bacterial reads, followed by *Actinobacteria*, *Planctomycetes*, *Bacteroidia*, and *Acidobacteria* (22 ± 7, 6 ± 3.6, 5.4 ± 1.7, and 4.4 ± 3.9% of bacterial reads, respectively). Fungal decomposers accounted for more than half of the eukaryotic reads (58.4 ± 15.1%). The great majority belonged to *Ascomycota* (88.1 ± 24% of fungal reads), followed by *Basidiomycota* (6.8 ± 2.1% of fungal reads). However, 18 ± 1% of the fungal reads were identified as yeast-forming taxa like *Candida* (15 ± 3.8% of fungal reads). Only a minor proportion of potential plant pathogens (7%) were detected among fungi. Consumers were dominated by protists, which accounted for 8.4 ± 2.6% of eukaryotic reads, predominantly the amoebozoan classes *Myxogastria* and *Variosea* (42.5 ± 24.6 and 5.5 ± 1.6% of protistan reads, respectively), and ciliates in *Colpodea* and cercozoans in *Sarcomonadea* (22.2 ± 9.5 and 5.3 ± 2.4% of protistan reads, respectively). The genera *Licea* and *Colpoda* together accounted for almost half of the community of protists (24.5 ± 21.3 and 22.2 ± 9.5% of protistan reads, respectively). Microscopic metazoans accounted for 1.5 ± 0.7% of eukaryotic reads and were dominated by *Rotifera* and *Tardigrada* (50.6 ± 27 and 32.9 ± 27% of metazoan reads, respectively), with the tardigrade genus *Ramazzottius* accounting for more than one-quarter of the total metazoan community. The remaining microscopic metazoans were nematodes with 16.5 ± 13.3% reads at low frequency. Predatory bacteria occurred only in low abundance (2.3 ± 1.1% of bacterial reads) and were dominated by *Myxococcota* (93.6 ± 3.2% of predatory bacteria reads).

### Core co-occurrence network analyses of the microbial food web

**Figure 2:**
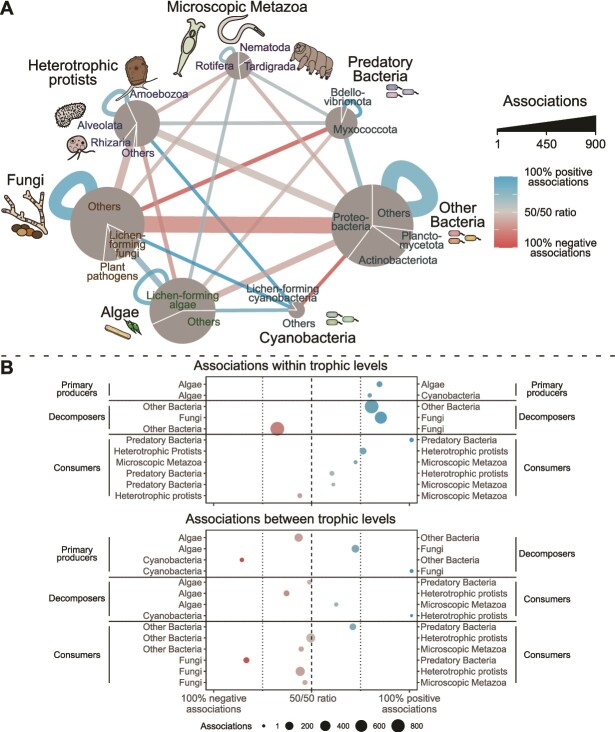
**Summarized core co-occurrence networks of the microbial community in tree canopies**. The core co-occurrence network (A) shows associations between genera on the bark surfaces in tree canopies (N = 15). The associations are summarized for different taxonomic groups, i.e. for algae and cyanobacteria (primary producers), fungi and other bacteria (decomposers), and predatory bacteria, heterotrophic protists, and microscopic metazoans (consumers). The node size is proportional to the log-transformed relative number of reads for bacteria and eukaryotes respectively. The thickness of the edges indicates the number of associations between taxonomic groups and the color code for the ratio of negative to positive associations. A summary of the network as a dot plot (B) shows the ratio of negative to positive associations with the size of the points corresponding to the number of associations between and within trophic levels.

Network analysis across all tree species revealed a complex core microbial food web composed of 579 genera with 3936 associations (Fig. 2, Supplementary Fig. 3). The food web was dominated by bacterial and fungal decomposers (247 and 198 genera, respectively). Additionally, we found a high proportion of lichen-forming genera (57% of algal, 15% of cyanobacterial, and 13% of fungal reads) such as *Chloroidium*, *Trebouxia*, and *Apatococcus* (the three dominant algal genera), *Gloeocapsa* (cyanobacteria), as well as *Physcia*, *Lecanora*, and *Lecidea* (fungi). Moreover, also a few associations between previously described lichen symbionts, such as between *Chlorella* and *Trapeliopsis*, were detected.

Most associations (67.2%) were found within trophic levels, and the majority (64.1%) of these associations were positive. Outstanding, however, was the high proportion of negative associations (68.6%) between bacterial and fungal decomposers. Between trophic levels, fungi showed primarily positive associations with cyanobacteria and algae (100% and 71.3% of associations, respectively), indicating potential symbiotic relationships. In contrast to fungi, bacteria showed highly negative associations with cyanobacteria and algae (86.7% and 57.7% of associations, respectively). Moreover, 68.8% of all associations between trophic levels occurred between consumers, i.e. protists, microscopic metazoans and predatory bacteria, and their potential prey. Predatory bacteria (*Myxobacteria*, *Bdellovibrio*) exhibited mainly positive associations with bacterial decomposers (70% of associations) but mainly negative associations with fungal decomposers (84.4% of associations), whereas protists and microscopic metazoans did not show clear patterns in the ratio of negative to positive associations.

### Differences of bark microbiomes between the tree species

**Figure 3:**
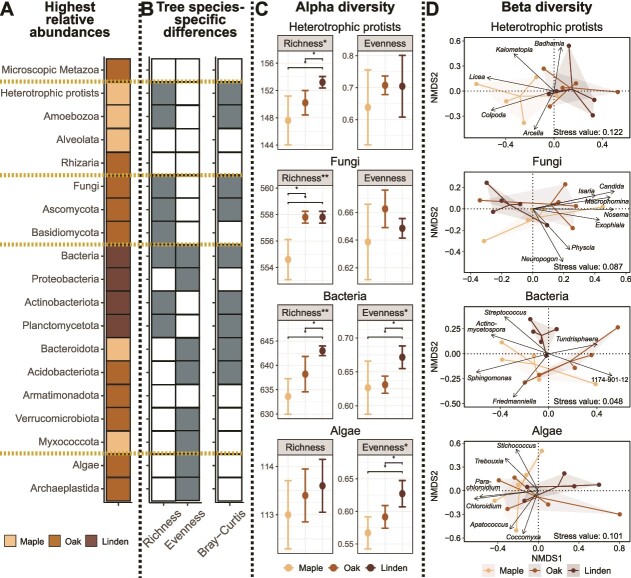
**Tree species-specific differences.** Heat map for selected microbial groups showing (A) the highest relative abundance of specific microbial taxa on the respective tree species and (B) differences in genus richness and Pielou’s evenness (alpha diversity) as well as in Bray-Curtis dissimilarities (beta diversity) between the tree species (N = 5). Grey-colored areas indicate significant differences (alpha diversity = Kruskal–Wallis test, Bata diversity = PERMANOVA, *P* value <0.05). Dot plots (C) show the alpha diversity indices per tree species (mean ± 1 SD). Significant differences across all tree species (Kruskal–Wallis test) are marked in the graph title, significant differences for pairwise comparisons of tree species (Wilcoxon test) are marked in the graph with stars (^*^P value <0.05; ^*^^*^P value <0.01; ^*^^*^^*^P value <0.001). Non-metric multidimensional scaling of Bray–Curtis dissimilarity (D) show the multivariate dispersion of the samples. Lines are color-coded by tree species and link samples of each tree species to their centromere. The relative abundances of the 10 most abundant genera classified to genus level were correlated onto the respective ordinations, significant correlations (*P* value <0.05) are shown as arrows.

A comparison of the relative abundance, alpha, and beta diversity of the bark microbiomes revealed significant differences between maple, oak, and linden (Fig. 3, Supplementary Fig. 4-6). However, these differences were not uniform but strongly differed between microbial groups.

Maple accommodated a depauperate microbiome, with a significantly lower genus richness of bacteria (*Actinobacteriota*, *Planctomycota*), fungi and protists (*Amoebozoa*). Still, maple showed the highest relative abundance of the bacterial phyla *Bacteroidota*, *Myxococcota* (Fig. 3A), and the genera *Sphingomonas* and *Actinomycetospora* (Fig. 3D). Likewise, among eukaryotes, the algal genera *Coccomyxa*, *Chloridium*, *Parachloroidium*, and *Stichococcus* (Fig. 3D)*,* as well as the protistan genera *Licea* (*Amoebozoa*) and *Colpoda* (*Alveloata*) (Fig. 3A, 3D) dominated on maple. This resulted in a low evenness of algae and bacteria on maple (Fig. 3B & C) and a distinct community composition of protists compared to the other tree species (Fig. 3B & D, Supplementary Fig. 4).

The oak microbiome showed the highest relative abundance of algae and fungi, *Rhizaria* (protists), and microscopic metazoans (Fig. 3A). Many lichen-forming algae such as *Apatococcus, Dictyochloropsis*, *Trebouxia*, and *Trentepohlia* together with lichen-forming fungi such as *Physcia* and yeast cells (*Candida*) showed their highest relative abundance on oak (Fig. 3D), as well as microscopic metazoans in *Adineta*, *Rotaria* (*Rotifera*), *Ramazzottius* (*Tardigrada*), and fungivorous *Laimaphelenchus* (*Nematoda*). In terms of genus richness, oak exhibited an intermediate genus richness between maple and linden (Fig. 3B & C). Similarly, the genus evenness of algae and bacteria was higher on oak than on maple but lower than on linden (Fig. 3B & C). However, the oak microbiome exhibited no distinct community composition in terms of beta diversity (Fig. 3B & D, Supplementary Fig. 4).

The linden microbiome showed the highest relative abundance of bacteria, with the dominant phyla *Proteobacteria*, *Actinobacteriota*, and *Planctomycetota* (Fig. 3A), along with the highest genus richness of bacteria and protists (Fig. 3C). Additionally, with the highest evenness of algae and bacteria, linden bark hosted quite diverse communities of primary producers and decomposers (Fig. 3C). The composition of bacterial and fungal communities on linden differed significantly from maple and oak (Fig. 3D, Supplementary Fig. 4).

## Discussion

### Bark microbiome

Our comprehensive analysis revealed a diverse bark microbiome in tree canopies, comprising nearly 1500 genera. Quantitatively, our method revealed its strengths in uncovering a substantially higher diversity of eukaryotes compared to earlier amplicon sequencing studies on bark microbiomes [e.g. 9, 48–50], in particular of algae and protists. Qualitatively, our findings correspond well with data obtained by light microscopy and culture-based studies [e.g. 51–54].

Tree bark surfaces typically constitute sunlight-exposed but water and nutrient-limited habitats for microorganisms. Primary producers form the basis of the microbial food web. Their ability to utilize sunlight for carbon fixation forms an important nutrient and energy source for heterotrophic organisms [3, 7, 55]. Algae were by far the most diverse and dominant primary producers on bark with 113 genera in 28 classes, whereas cyanobacteria contributed only a small proportion. We confirmed the prevalence of *Chlorophyta* [48–50], especially the *Trebouxiophyceae*, which comprise spherical or ellipsoid solitary algae, such as *Chloroidium* and *Trebouxia*, and sarcinoid colony-forming algae, like *Apatococcus* [14, 52]. In addition, we found a high abundance of uniseriate filament-forming algae such as *Trentepohlia* (*Trentepohliales*), one of the most widespread terrestrial algae [56].

The decomposer communities on bark were dominated by bacteria with 644 genera in 52 classes, whereas *Archaea* were rather scarce. The general prevalence of *Alphaproteobacteria*, *Actinobacteria*, *Bacteroidia*, and *Acidobacteria* corresponds to previous amplicon sequencing and metagenomics studies [9, 12, 48, 49, 57]. With almost 10%, the alphaproteobacterium *Sphingomonas* was especially abundant in our bacterial reads. *Sphingomonas* is a well-known inhabitant of bark surfaces [e.g. 9, 48] and may promote plant growth and resistance by producing various phytohormones and antifungal compounds [58, 59]. Fungi were dominated by *Ascomycota* comprising 88% of fungal reads. Among yeasts *Candida* (15% of fungal reads) reached an exceptionally high relative abundance, a result also sustained by culture-based studies [51, 60]. The ability of *Candida* yeasts to degrade cellulose and hemicelluloses may favor its presence on bark [61].

Among consumers, protists were by far the most diverse group with 155 genera in 33 classes. A particular strength of our metatranscriptomic study is that it allows a comprehensive comparison of the dominance of protistan taxa and their life strategies in tree canopies. The protists were dominated by *Amoebozoa*, which are rarely detected and grossly underestimated by DNA-based methods due to their inherent high sequence variability, and variable sequence length of the V4 barcoding region in the 18S ribosomal RNA gene [16, 62]. Our RNA-based approach instead reliably identified a high richness of the amoebozoan class *Myxogastria* with various corticolous (bark-loving) genera such as *Licea*, *Arcyria*, *Physarum*, and *Echinostelium* [54, 63], which confirms the findings of microscopic/culture-based studies [54, 63]. The corticolous *Variosea* and *Myxogastria* are particularly well adapted to the variable life conditions on bark surfaces, as they can form resistant cysts, sporocarps, and spores within hours at declining moisture conditions [64, 65]. Other protists appear particularly predisposed for life on bark surfaces as they can complete their whole life cycle in just a few hours. This is true for flagellates and amoeboflagellates in *Sarcomonadea* (*Cercozoa*) [66] and in particular for *Colpoda* (*Colpodea*) species among ciliates. Early light-microscopic investigations of protists on plant surfaces revealed a prevalence of *Colpoda cucullus*, a ciliate with an impressively short life cycle due to cell division within drought-resistant resting cysts [67, 68]. In our study, *Colpodea* clearly dominated the ciliate community, however, aside from the dominant *Colpodea*, a considerable number of other tree-adapted species was assessed by previous studies [53, 69]. Another adaptation to life on bark was detected by comparing communities of canopy protists with those in the litter layer and soil on the ground [70]. Apart from the dominance of small r-strategists among protists discussed above, a striking prevalence of testate amoebae was described [70], whose shells may prolong their foraging times due to enhanced protection against drought. Molecular studies of cercozoan diversity in tree canopies of the Leipzig floodplain forest [13] confirmed the prevalence of drought-resistant testate amoeba (e.g. *Thecofilosea*) as well as of *Sarcomonadea* with rapid life cycles; a pattern well-reflected in our data (Fig. 1, Supplementary Fig. 2). However, protists are not the only consumers in microbial food webs. Recent studies highlighted predatory bacteria as one of the key consumers of bacteria in soil [71, 72]. According to the low proportion of predatory bacteria in our data, they appear to be of much lower importance on bark. Nematodes were quite rare, but dominated by the genus *Laimaphelenchus*, common colonizers of tree bark, known feed on algae, mosses and lichen [73]. Instead, the microfauna on trees was dominated by *Ramazzottius*, a widespread tardigrade genus [74], accounting for more than a quarter of the metazoan community.

The soundness of the metatranscriptomics data to results obtained by direct microscopic estimations of protistan diversity, the reliable detection of groups commonly isolated from bark that evade detection by general eukaryotic primers [17, 18], and coherence to the diversity obtained at much greater sequencing depth with taxon-specific primers [e.g. 13], provide strong evidence for the reliability of this method to reflect the true diversity of the bark microbiome.

### Core microbial food web

The ubiquitous taxa across the three tree species comprised the core microbial food web. It was composed of 579 distinct genera with a great richness of bacterial (247) and fungal (198) genera among the decomposers, 51 genera of primary producers and 83 genera of consumers.

The network analysis indicates that lichen symbioses are a characteristic feature of the core bark microbiome. Among the primary producers, almost 60% of algal reads and 15% of cyanobacterial reads could be assigned to potential lichen symbionts (photobionts). The dominant *Trebouxia* (11.7% of all algal reads) is known as the most common algal lichen symbiont [32, 75]. Correspondingly, 13% of the fungal reads were assigned to typical lichen-forming taxa (mycobionts) such as *Physcia*, *Lecanora*, and *Lecidea* [76, 77]. In addition, 71.3% of algae-fungi associations were positive. These even include some associations between previously described lichen partners such as *Chlorella* and *Trapeliopsis* [32] although identifying lichen partners is challenging, as more and more sequencing studies reveal unsuspected promiscuity among fungi and algal partners in lichen symbioses [78–80]. The strong positive associations between algae and fungi and the high proportion of lichen symbionts, despite the strict avoidance of sampling any visible lichens, indicate widespread early stages of symbioses, e.g. loosely associated cells, hyphal webs or propagules [32, 81, 82].

The high proportion of negative associations of bacteria with algae and fungi indicates an antagonistic relationship. In contrast to aquatic systems, where bacteria are the main beneficiates of phytoplankton exudates and where symbiotic interactions between algae and bacteria are widespread [83, 84], bacteria compete for algal exudates with fungi on bark. Fungal symbioses are likely favored by the dry conditions on bark [85], and may be further enhanced by antibacterial metabolites of algae and fungi [82, 86]. *Vice versa*, *Actinobacteria* and *Sphingomonas* which dominated the bacterial bark community, are known for producing antifungal metabolites [59, 87, 88]. This again may feed back on potential plant pathogenic fungi or oomycetes [5, 89], which were only detected at low frequency (fungi) or absent (oomycetes).

The bark food web contained a substantial proportion of consumers at higher trophic levels. Assuming a gross growth efficiency of 30–50% [90], the higher trophic levels on the tree bark must be sustained by a substantially larger proportion of primary, bacterial, and fungal production. Protists are the primary consumers in our food web (8.4% of eukaryotic reads), followed by predatory bacteria (2.3% of bacterial reads) and microscopic metazoans (1.5% of eukaryotic reads). Many protists and metazoans of the phyllo- and rhizosphere are omnivores and consume a broad range of algae, bacteria, and fungi [91–93], which is reflected by the numerous associations between these groups in the network analysis. Most of the protists and microscopic metazoans did not show any clear directional ratio of negative to positive associations, as can be expected from omnivores with frequent prey shifts. An exception was *Colpodea* (*Colpoda* and *Exocolpoda*) exhibiting numerous associations with different bacterial taxa, including strongly positive associations with cyanobacteria. In agreement with previous studies [71, 72], predatory bacteria exhibited strong positive co-occurrence with bacterial decomposers in our network analyses. Predatory bacteria also exhibited strong co-exclusion with fungi that might be partly explained by their ability to inhibit fungal growth [72], but more likely reflects an indirect association driven by the co-exclusion of bacterial and fungal decomposers on bark.

### Tree species-specific differences of their bark microbiomes

We hypothesized that trees with rough bark (linden and oak) would host more similar microbiomes as compared to trees with smooth bark (maple). Tree species-specific differences have so far been shown for specific microbial groups on bark surfaces [9, 11, 12, 14, 15, 65, 94], but not for the entire microbiome. In this study, we confirmed tree species-specific differences among the bark microbiomes, driven by different microbial groups.

Maple with smooth bark accommodated a poorer microbiome, characterized by low genus richness and evenness, compared to oak and linden with rough bark. Beta diversity patterns showed an increased relative abundance of (i) desiccation-tolerant genera such as the bacterial *Sphingomonas* and the algae *Chloroidium*, *Trebouxia*, *Stichococcus*, and *Coccomyxa* [14, 52, 95, 96]; and (ii) UV protected taxa such as the pigmented bacteria in *Chitinophagaceae* [97] and eukaryotes in *Myxogastria* [98]. The smooth maple bark may provide less protection against environmental stressors like UV radiation or desiccation, thus supporting a sparser microbiome. However, tree species-specific differences in the composition of canopy microbiomes, could only partly be explained by bark topology. Whereas differences between smooth and rough bark communities were mainly driven by genus richness and evenness, microbial groups on oak and linden with rough bark differed mostly in their relative proportions. Oak was characterized by a higher relative abundance of algae and fungi, including many lichen-forming genera. Linden was characterized by a higher relative abundance of bacteria. Furthermore, microbial communities on linden exhibited the highest evenness and a distinct community composition in terms of beta diversity. These differences in evenness and beta diversity were mainly driven by primary producers and decomposers suggesting that they are stronger shaped by selective pressures than higher trophic levels.

### Concluding remarks

The shotgun metatranscriptomic approach allowed the simultaneous assessment and thus comparison of the entire living prokaryotic and eukaryotic microbial canopy bark communities among oak, linden and maple. We identified highly diverse and tree species-specific differences in the composition of canopy microbiomes, only partly explained by bark topology. Still, all tree species also harbored a joint and taxonomic diverse core microbiome. Strong algal-fungal co-occurrence indicated microscopic lichen symbioses. Also, the life-strategies of the dominant protistan taxa reflected a variety of specific adaptations to the harsh environmental conditions on bark. We detected strongly negative associations of algae and fungi with bacteria. Their consumers were myxobacteria, microscopic metazoans, and especially protists. The many omnivores among protists, also consuming algae, yeasts, and other protists, reflect more complex food-web interactions. Although potential plant pathogens were rare, the method allowed their assessment relative to the total microbial diversity, and network analysis showed great potential to identify likely microbial interdependencies. Understanding the microbiome dynamics is essential, as they impact host plant fitness, function, and productivity, thereby influencing tree health and ecosystem productivity.

## Supplementary Material

FreudenthalEtAl2024_BarkMicrobiome_ISME_FinalSupplement_wrae206

## Data Availability

The raw data are available under NCBI BioProject PRJNA1105877. The code for the presented analyses is available at GitHub: https://github.com/JFreude/BarkMicrobiome.
