# Supplementary material for: Algae-fungi symbioses and bacteria-fungi co-exclusion drive tree species-specific differences in canopy bark microbiomes
Source: ISME J. 2024 Oct 17;18(1):wrae206. doi: 10.1093/ismejo/wrae206 (PMC11630260; doi:10.1093/ismejo/wrae206)
Supplement: FreudenthalEtAl2024_BarkMicrobiome_ISME_FinalSupplement_wrae206 [file freudenthaletal2024_barkmicrobiome_isme_finalsupplement_wrae206.docx]

Supplementary information

Algae-fungi symbioses and bacteria-fungi co-exclusion drive tree species-specific differences in canopy bark microbiomes

**Authors**

Jule Freudenthal^a^, Kenneth Dumack^a^, Stefan Schaffer^b^, Martin Schlegel^c,d^ and Michael Bonkowski^a^

^a^ Terrestrial Ecology, Institute of Zoology, Cluster of Excellence on Plant Sciences (CEPLAS), University of Cologne, Zülpicher Str. 47b, 50674 Köln, Germany

^b^ Institute of Biology, Molecular Evolution and Systematics of Animals, University of Leipzig, Talstraße 33, 04103 Leipzig, Germany

^c^ Institute of Biology, Biodiversity and Evolution, University of Leipzig, Talstraße 33, 04103 Leipzig, Germany

^d^ German Centre for Integrative Biodiversity Research (iDiv) Halle Jena Leipzig, Puschstraße 4, 04103 Leipzig, Germany

**Corresponding authors:** Jule Freudenthal^1^ and Michael Bonkowski^2^

^1^ jule.freudenthal@uni-koeln.de

^2^ m.bonkowski@uni-koeln.de


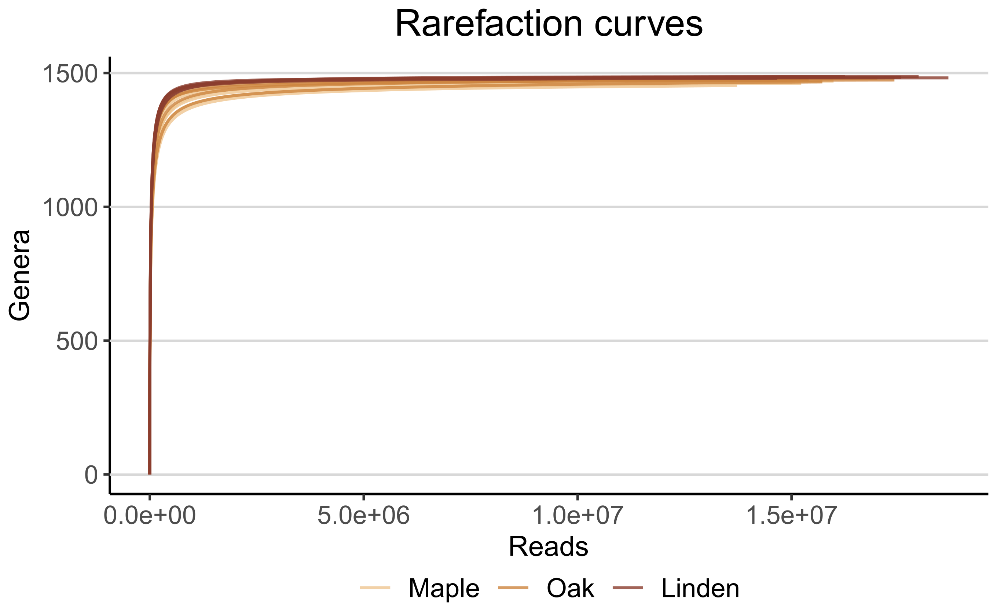


**Supplementary Figure 1: Rarefaction curves.** Rarefaction curves show the number of reads in relation to the number of genera (N=15 samples). All samples reached complete saturation. The samples are color-coded by the tree species.


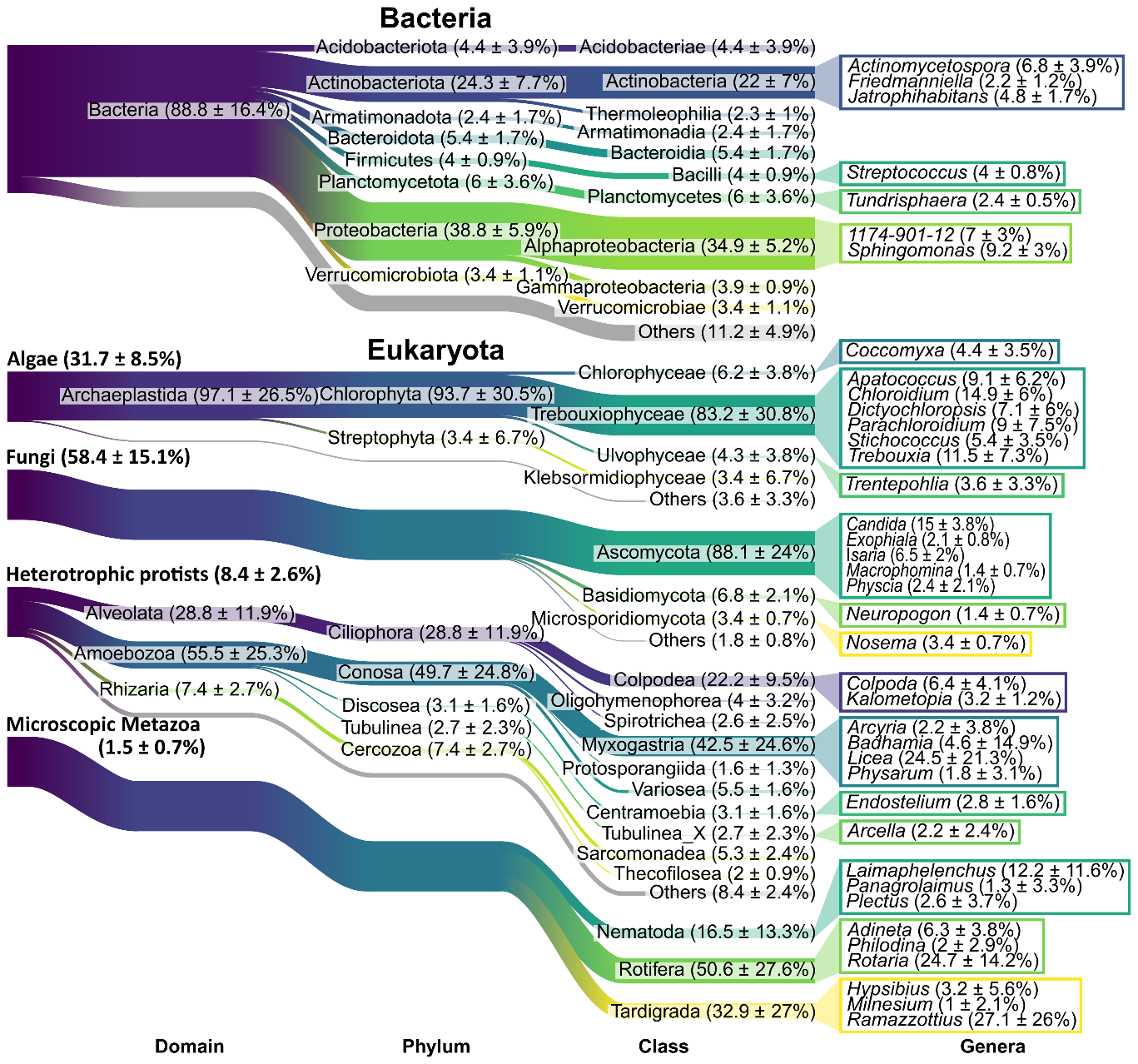


**Supplementary Figure 2:** **Microbial community composition in tree canopies.** Sankey diagrams show the mean relative abundance (percent) and standard deviation across all tree species (N=15) of the 10 most abundant classes of bacteria, algae, fungi, heterotrophic protists, and microscopic metazoa that accounted for more than 1% of the respective community. Additionally, the 10 most abundant genera that were classified to genus level are shown for each community, respectively.

**Supplementary Figure 3: Co-occurrence networks of the microbial community in tree canopies.** The co-occurrence networks show associations between genera found on the bark surfaces in tree canopies (N=15). The networks are arranged by different trophic levels with primary producers at the bottom, decomposers in the middle, and consumers at the top. Associations are shown between all genera (A), between the trophic levels (B), and within the trophic levels (C). The node size is proportional to the relative number of reads for bacteria and eukaryotes respectively. Edges represent negative (red) and positive (blue) associations between the genera.


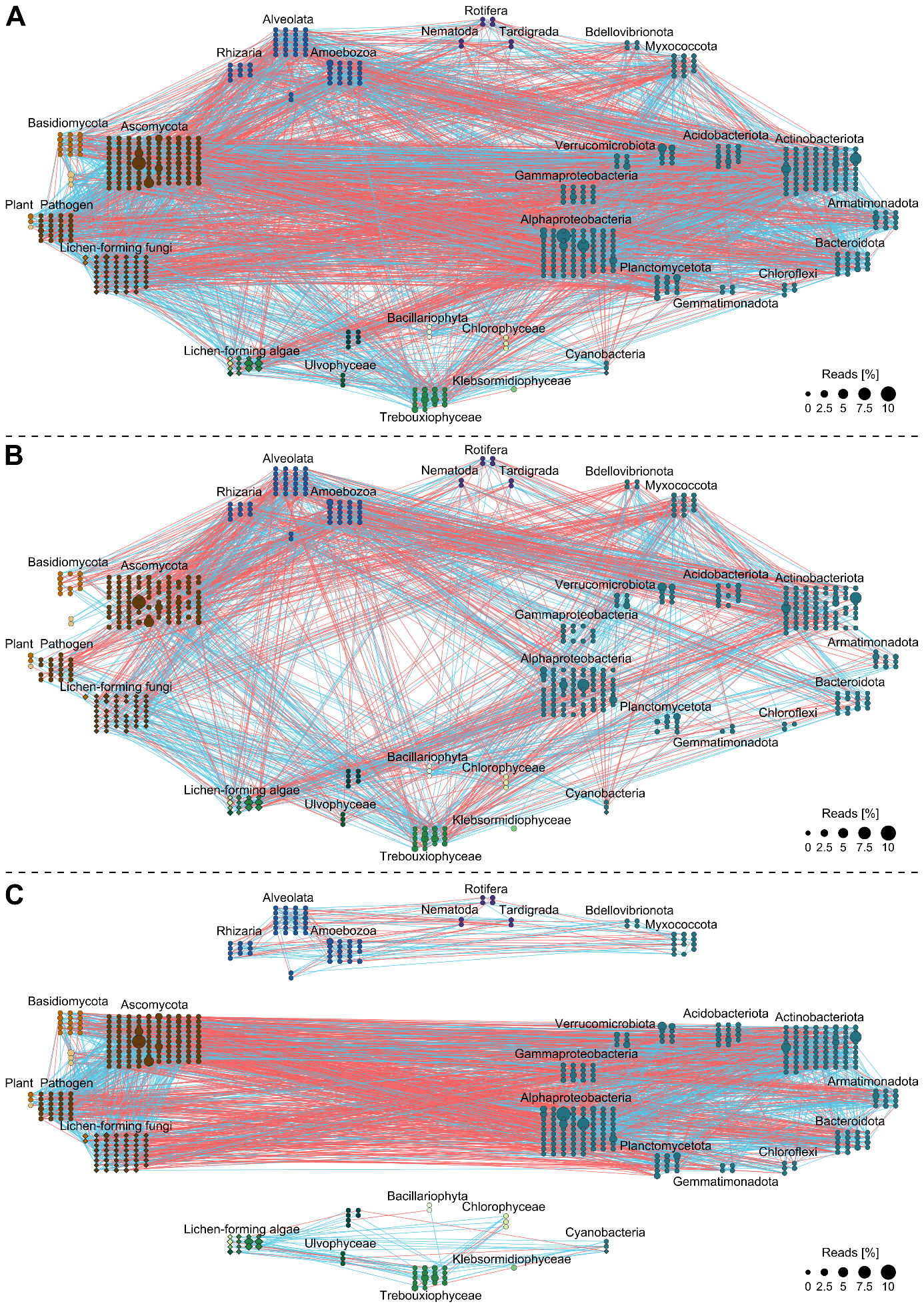

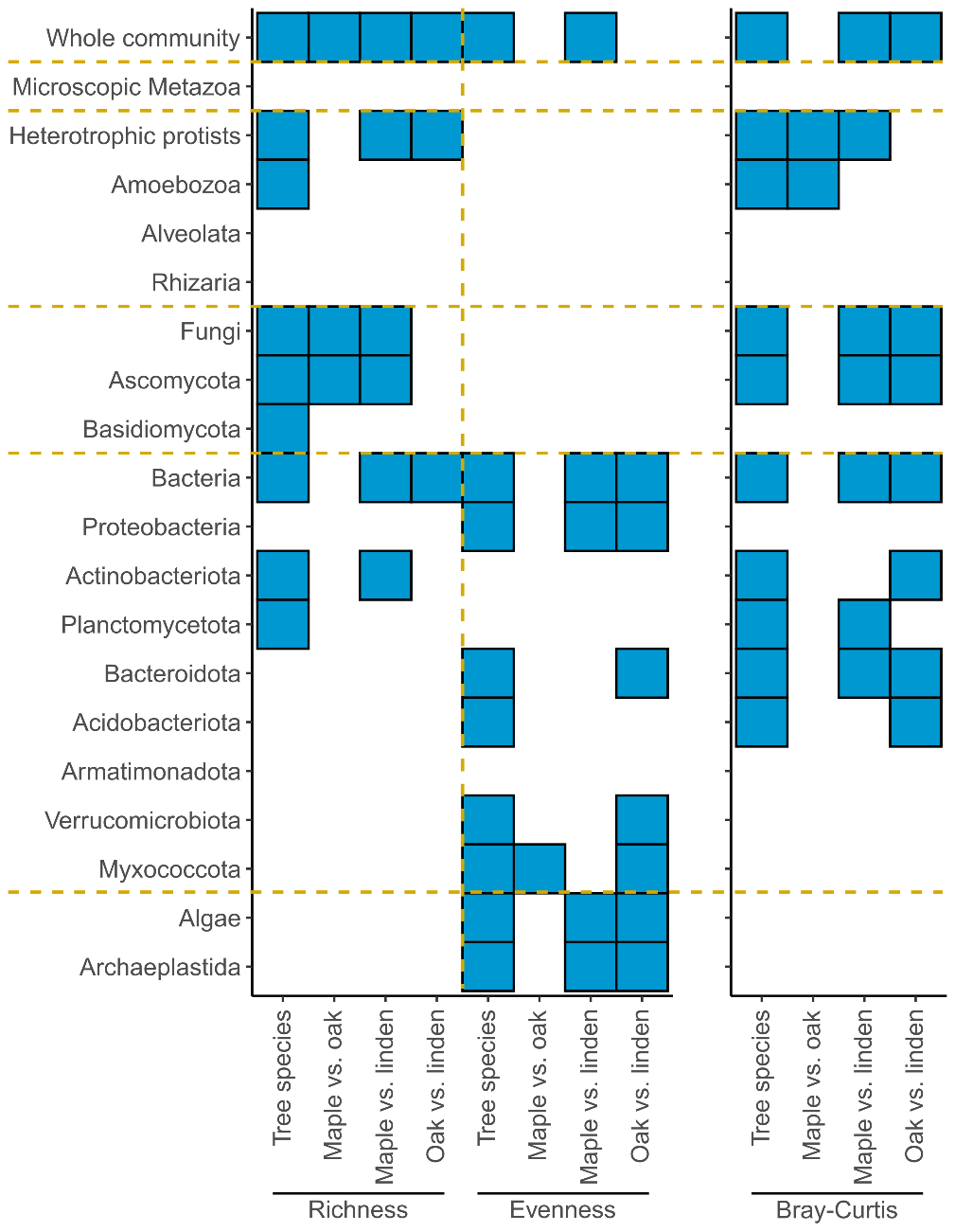


**Supplementary Figure 4:** **Overview of significant differences of tree species-specific alpha and beta diversity indices.** The heat map shows significant differences in genus richness and Pielou’s evenness as well as Bray-Curtis dissimilarities across all tree species (first column respectively, alpha diversity=Kruskal-Wallis test, beta diversity=perMANOVA) as well as differences in pairwise comparisons of tree species (alpha diversity=Wilcoxon test, beta diversity= Pairwise perMANOVA) for selected taxonomic groups. Blue colred areas indicate significant differences (*P* value < 0.05).

**Supplementary Figure 5: Overview of tree species-specific alpha diversity indices.** Dot plots show the genus richness and Pielou's evenness per tree species for selected taxonomic groups. The dots represent the mean value, the error bars the standard deviation. Significant differences across all tree species (Kruskal-Wallis test) are marked in the graph title significant differences for pairwise comparisons of tree species (Wilcoxon test) are marked in the graph with stars (* *P* value < 0.05; ** *P* value < 0.01; *** *P* value < 0.001).


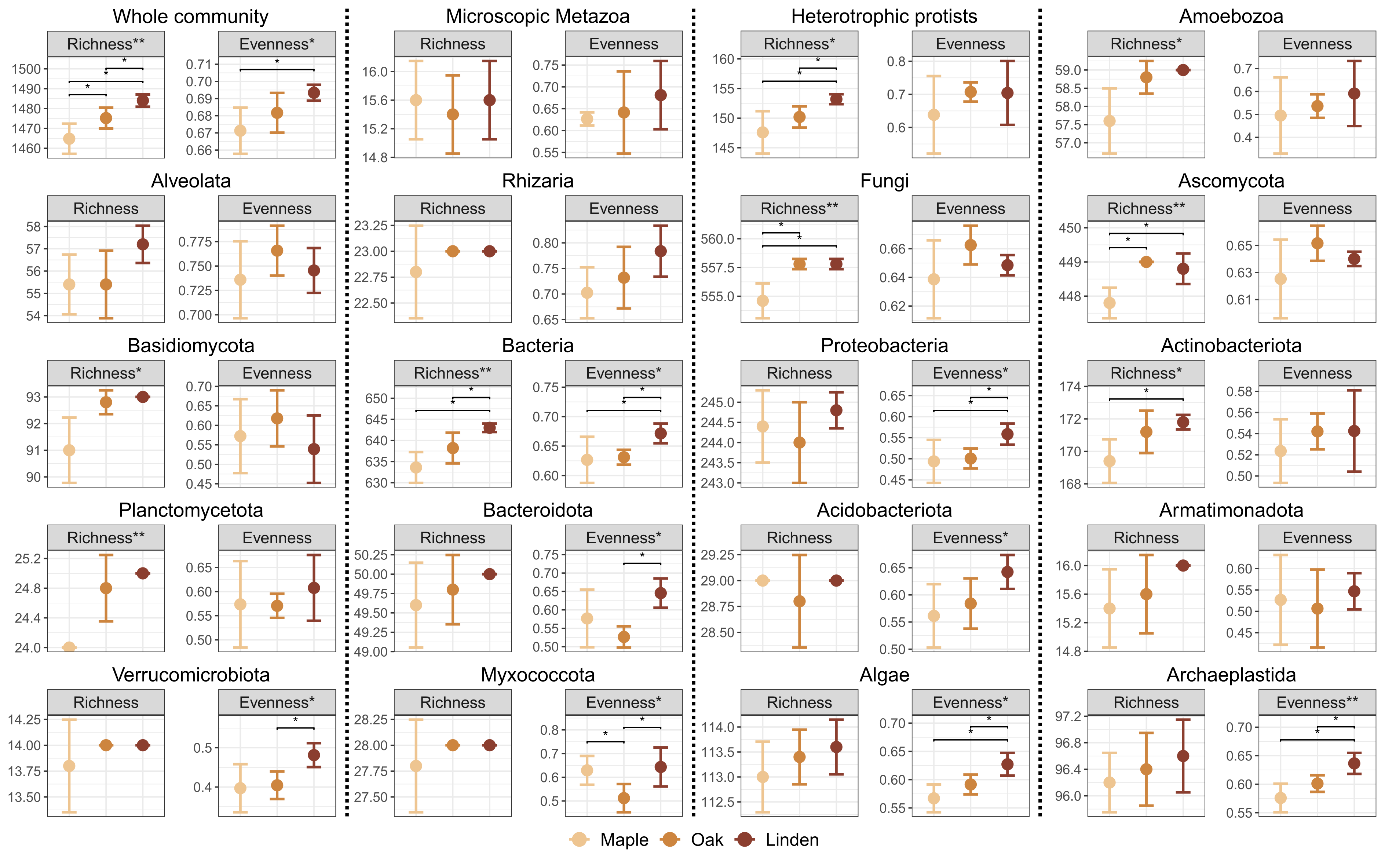


**Supplementary Figure 6: Overview of tree species-specific beta diversity.** Non-metric multidimensional scalings (NMDS) of Bray-Curtis dissimilarity show the multivariate dispersion of the samples for selected taxonomic groups. Lines are color-coded by tree species and link samples of each tree species to their centromere.


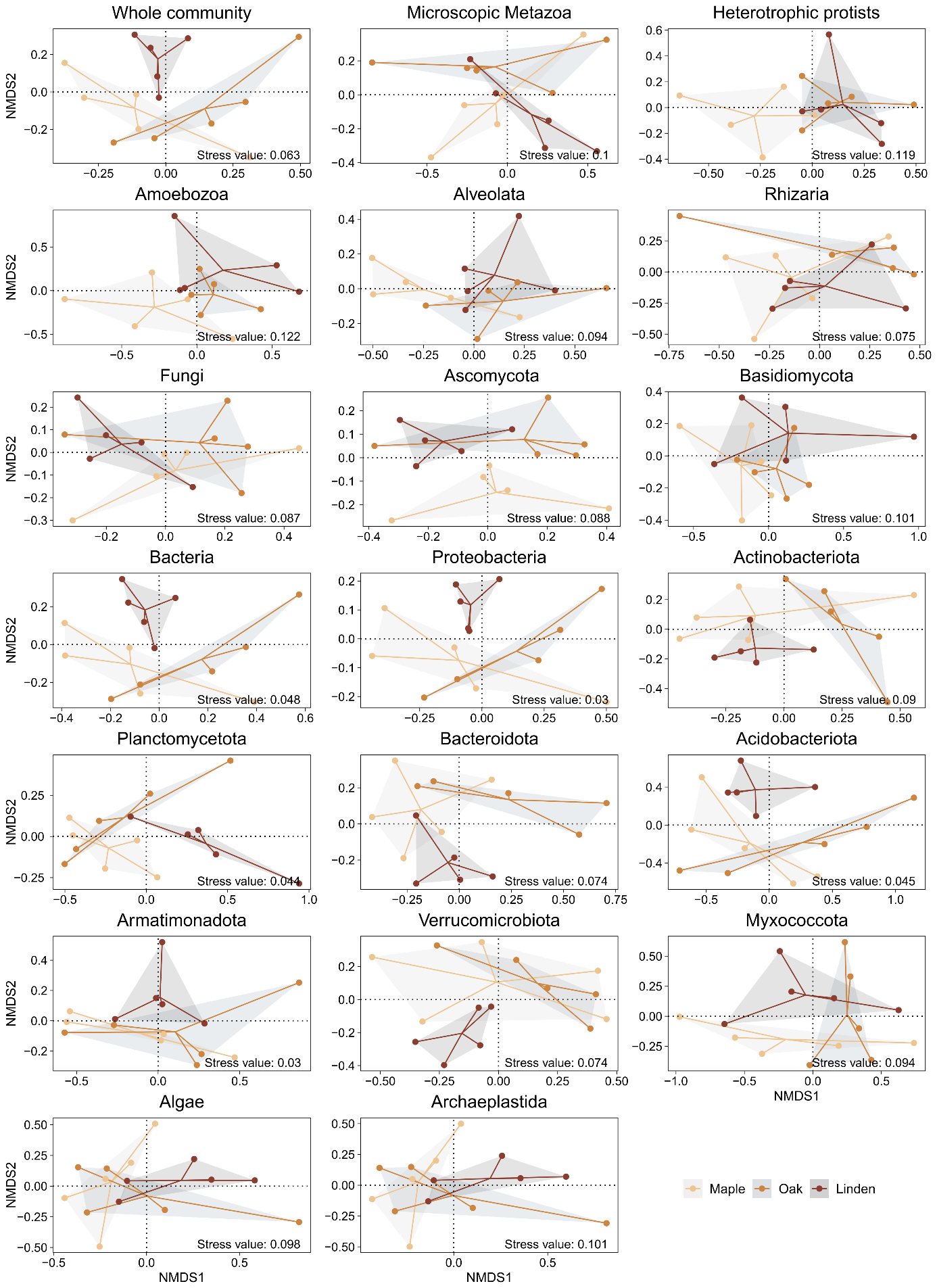


**Supplementary Table 1: Overview of the number of reads per processing step**. For each sample, the number of reads after quality filtering, the number of reads successfully assembled into contigs, and the average contig length are shown for each sample. In addition, the number of hits for the PR^2^ and Silva databases are shown.

| Tree | Replicate | Number of reads | | Number of contigs | Mean length of contigs | Database matches | | |
| --- | --- | --- | --- | --- | --- | --- | --- | --- |
|  |  | Total | Filtered |  |  | PR^2^ | | SILVA |
| Maple | 1 | 62'066'095 | 54'782'009 | 34'267'056 | 218 | 6'857'979 | 10'859'015 | |
| Maple | 2 | 61'182'230 | 54'834'717 | 30'029'067 | 230 | 6'617'627 | 9'902'565 | |
| Maple | 3 | 57'642'915 | 51'623'620 | 26'500'849 | 230 | 7'510'904 | 7'786'301 | |
| Maple | 4 | 68'321'722 | 60'934'697 | 31'625'283 | 232 | 7'256'503 | 9'636'821 | |
| Maple | 5 | 67'760'636 | 60'176'238 | 32'602'613 | 227 | 6'160'160 | 11'345'074 | |
| Oak | 1 | 63'551'723 | 57'268'788 | 33'384'234 | 228 | 9'073'024 | 9'934'790 | |
| Oak | 2 | 65'635'053 | 58'974'935 | 32'298'611 | 229 | 5'224'063 | 12'335'655 | |
| Oak | 3 | 64'953'704 | 58'186'565 | 34'978'135 | 226 | 9'861'618 | 9'500'957 | |
| Oak | 4 | 61'439'467 | 54'765'861 | 30'547'978 | 228 | 8'545'337 | 8'572'851 | |
| Oak | 5 | 66'993'556 | 60'139'618 | 27'541'468 | 236 | 7'182'410 | 8'003'008 | |
| Linden | 1 | 80'623'991 | 72'008'768 | 37'192'288 | 233 | 5'520'558 | 13'818'189 | |
| Linden | 2 | 63'944'549 | 57'229'574 | 29'761'371 | 232 | 6'919'860 | 9'201'105 | |
| Linden | 3 | 70'370'573 | 63'065'424 | 37'658'841 | 225 | 5'462'431 | 13'574'846 | |
| Linden | 4 | 75'151'907 | 66'997'536 | 38'528'179 | 229 | 8'390'810 | 12'738'239 | |
| Linden | 5 | 70'830'597 | 63'392'067 | 35'355'216 | 229 | 6'384'769 | 12'035'556 | |
